# Efficacy and safety of radiofrequency ablation versus laparoscopic hepatectomy for small hepatocellular carcinoma: A protocol for a randomized controlled trial

**DOI:** 10.1097/MD.0000000000023678

**Published:** 2021-01-08

**Authors:** Fuping Zhu, Qing Chang, Shigang Duan, Weiye Leng

**Affiliations:** Department of General Surgery, The Ninth People's Hospital of Chongqing, Chongqing, China.

**Keywords:** laparoscopic hepatectomy, radiofrequency ablation, randomized controlled trial, small hepatocellular carcinoma

## Abstract

**Background:**

Hepatocellular carcinoma (HCC) is one of the most common type of malignant tumors in adults, and is the most common cause of death in people with cirrhosis. Both laparoscopic hepatectomy (LH) and radiofrequency ablation (RFA) are radical treatments for small HCC. However, there is no international standard for the treatment of small HCC, and it is still controversial to choose LH or RFA in treating small HCC. We try to carry out a randomized, controlled, prospective study to compare the the short-term and long-term effects and safety of LH versus RFA in the treatment of small HCC.

**Methods:**

This study is a single-center, evaluator-blinded, randomized, controlled clinical trial (RCT). The patients will be randomly divided into RFA group and LH group in a 1:1 ratio according to a computer-generated randomization list. Postoperative complications rates, Alpha fetoprotein (AFP), hospital stay, 1, 2, 3-year overall survival (OS) rates, disease-free survival (DFS) rates and all possible adverse events will be recorded. Statistical analyses will be performed with SPSS v22.0 software.

**Conclusions:**

The study will compare the the short-term and long-term effects and safety of LH versus RFA in the treatment of small HCC.

**OSF Registration number:**

doi: 10.17605/OSF.IO/HNX2T

## 1 Introduction

Hepatocellular carcinoma (HCC) is one of the most common type of malignant tumors in adults, and is the most common cause of death in people with cirrhosis.^[1,2]^ The vast majority of HCC occurs in Asia and sub-Saharan A, especially in China, with an incidence rate of 21.35 per 100,000 and a case fatality rate of 18.43 per 100,000.^[3]^ It occurs in the setting of chronic liver inflammation, and is mainly related to hepatitis B virus infection, liver cirrhosis, or exposure to alcohol or aflatoxin.^[4]^ Small HCC refers to a single cancer nodule with a maximum diameter of ≤3 cm or two nodules with a combined diameter of ≤3 cm, without obvious symptoms and signs.

Surgical resection is associated with better cancer prognosis. The conventional open hepatectomy is associated with more blood loss, longer recovery time, high incidence of complication and mortality.^[5]^ With the improvement of laparoscopy technology, laparoscopic hepatectomy (LH) has been widely used in treating resectable HCC, with satisfactory short-term and long-term outcomes.^[6–8]^ However, less than 30% of patients with small HCC are eligible for surgery,^[9]^ due to many factors such as extrahepatic metastasis, vascular invasion, high-risk anatomical locations, excessive lesions, and insufficient liver to sustain life. Therefore, many non-surgical alternatives have been developed, including radiofrequency ablation (RFA). RFA has been established as the standard of care for patients with small HCC unsuitable for surgical resection. A large number of studies have shown that it is safe, effective, minimally invasive, with minimal complication and mortality.^[10–12]^

Both LH and RFA are radical treatments for small HCC. However, there is no international standard for the treatment of small HCC, and it is still controversial to choose LH or RFA in treating small HCC.^[13]^ Though RFA in safer than LH, the recurrence rate and local lesion progression rate are higher,^[14]^ which made LH the preferred treatment for HCC. Therefore, we try to carry out a randomized, controlled, prospective study to compare the the short-term and long-term effects and safety of LH versus RFA in the treatment of small HCC.

## 2 Method

### 2.1 Study design

**Figure 1:**
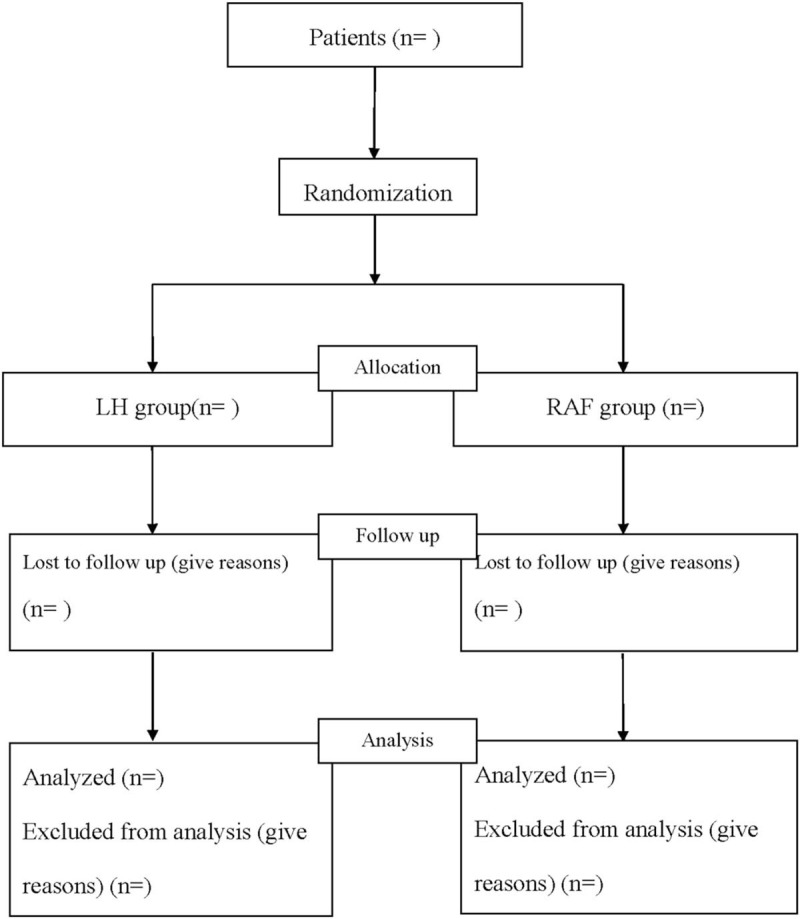
Flow diagram.

This study is a single-center, evaluator-blinded, randomized, controlled clinical trial (RCT), which has been registered in in open science framework (OSF) (Registration number: doi: 10.17605/OSF.IO/HNX2T). The protocol was approved by the Ethics Committee of The Ninth People's Hospital of Chongqing, and it will be carried out in accordance with the Declaration of Helsinki. The study protocol conforms to the Standard Protocol Recommendations for Interventional Trials (SPIRIT) 2013 Statement,^[15]^ and the results will be reported according to the CONSORT Statement extension for trials^[16]^ (Fig. 1).

### 2.2 Participants

#### 2.2.1 Sample size

Patients meeting the inclusion and exclusion criteria will be mainly recruited from inpatients of the Department of General Surgery, The Ninth People's Hospital of Chongqing.

According to the previous report, the 3-Year survival rate of small HCC is 70%, taking α = 0.05 and 1-β = 0.8, 355 samples will be needed in each group. Considering the withdrawal rate of 10%, the total number of patients is determined as 760, 380 in each group.

#### 2.2.2 Inclusion criteria

Patients will be included if they meet the following inclusion criteria:

(1) Patients meeting the diagnostic criteria for HCC;
(2) Child–Pugh class A or B;
(3) Three or fewer tumors ≤ 3 cm in diameter;
(4) No vascular invasion and no extrahepatic metastasis.
(5) Written informed consent.

#### 2.2.3 Exclusion criteria

Patients will be excluded with the following exclusion criteria:

(1) A total bilirubin ≥ 3.0 mg/dl;
(2) Platelet count < 40,000/μl or prothrombin activity < 40%;
(3) Decompensated liver cirrhosis, severe portal hypertension, history of bleeding from gastric fundus or esophageal varices, severe hypersplenism or refractory ascites;
(4) Presence of other uncontrollable malignancie.

#### 2.2.4 Randomization and blinding

The patients will be randomly divided into RFA group and LH group in a 1:1 ratio according to a computer-generated randomization list. Due to the limitation of treatment, the surgeons and patients are not blinded, and the statistical analysis will be carried out by an independent professional researcher who will not know the identification of the groups.

### 2.3 Interventions

All patients will be admitted to the hospital, and general information such as age and gender will be recorded. Before the operation, blood routine test, liver and kidney function, blood glucose, coagulation electrocardiogram, chest X-ray examination, and abdominal ultrasound, colonoscopy, pathological biopsy, will be examined if necessary, to exclude surgical contraindications.

Patients in LH group will get the laparoscopic liver resection treatment in general anesthesia, and each operation is performed by a surgeon with 10 years or more experience in laparoscopic liver resection. Anti-inflammatory, acid suppression, liver protection, maintenance of internal environment stability and symptomatic treatment will be performed after the operation.

Patients in RFA group will be treated with percutaneous radiofrequency ablation with Cool-tip, RF Ablation System (COVIDIEN) in local anesthesia. During the ablation process, each patient is injected intravenously with mida saliva to relieve pain. Anti-inflammatory, acid suppression, liver protection, maintenance of internal environment stability and symptomatic treatment will be performed after the operation.

### 2.4 Outcome variables

#### 2.4.1 Short-term outcomes

(1) Postoperative complications rates, involving high fever, biliary tract injury, bleeding, abdominal infection;
(2) Alpha fetoprotein (AFP) 2 months after operation;
(3) Hospital stay: the interval from the date of initial treatment to discharge.

#### 2.4.2 Long-term outcomes

(1) 1, 2, 3-year overall survival (OS) rates;
(2) 1, 2, 3-year disease-free survival (DFS) rates;
(3) Local recurrence rate.

Long-term outcomes will be recorded every 3 months during the follow-up visits to monitor survival rates and tumor recurrence. Follow-up visits will be closed at the time of death or the last visit of the patient.

#### 2.4.3 Safety evaluation

All possible adverse events (AEs) will be recorded, such as bleeding, pain, urinary retention.

### 2.5 Statistical methods

Statistical analyses will be performed with SPSS v22.0 software (SPSS Inc., Chicago, IL). Statistical testing is two-sided and *P* < .05 is considered to be statistically significant. Considering the homogeneity, group *t* test for normally distributed data or Mann-Whitney *U* test for non-normally distributed data will be used to compare variables between groups. A Pearson Chi-square test was applied to analyze categorical data. All curves were generated by the Kaplan-Meier method and the differences between the groups were compared using the log-rank test.

## 3 Discussion

At present, there is still no clear consensus on LH and RFA against small HCC.^[17]^ Surgical resection (SR) is supposed to be the standard treatment for HCC according to the Practice Guidelines of American Association For The Study Of Liver Diseases.^[18]^ However, they also suggested that SR is not recommended when the expected operative mortality rate of SR is greater than 3%, and other alternative methods such as RFA could be considered as another main treatment.^[19]^ RFA can improve the survival rate of patients, which made it the standard of care for patients with small HCC unsuitable for surgical resection.^[20]^ The RFA technique could avoid liver function damage, with a low complication rate. Therefore, we try to conduct a RCT study to compare the the short-term, long-term effects and safety of LH versus RFA in the treatment of small HCC.

However, this protocol could be criticized for the following limitations. First, certain biases might exist due to this study cannot be double-blinded because of the different operation methods. Second, it is a single-center study, and the source of patients is relatively limited, which may affect the conclusions to a certain extent. Third, it has been reported that the combination of transcatheter arterial chemoembolization with LH or RFA would greatly improve the curative effects of HCC,^[21]^ and further exploration is needed to prove that.

## Author contributions

**Funding acquisition:** Weiye Leng.

**Investigation**: Fuping Zhu and Qing Chang

**Project administration:** Fuping Zhu, Qing Chang.

**Study design**: Fuping Zhu and Qing Chang

**Supervision**: Shigang Duan

**Supervision:** Shigang Duan.

**Validation**: Weiye Leng

**Writing – original draft:** Fuping Zhu, Qing Chang.

**Writing – review & editing:** Weiye Leng and Weiye Leng.
